# ﻿A new species of karst-dwelling bent-toed gecko of the *Cyrtodactylusintermedius* group (Squamata, Gekkonidae) from eastern Thailand and the phylogenetic placement of *C.intermedius*

**DOI:** 10.3897/zookeys.1211.122563

**Published:** 2024-09-03

**Authors:** Natee Ampai, Attapol Rujirawan, Siriporn Yodthong, Korkhwan Termprayoon, Bryan L. Stuart, Anchalee Aowphol

**Affiliations:** 1 Department of Biology, Faculty of Science, Srinakharinwirot University, Bangkok, 10110 Thailand; 2 Animal Systematics and Ecology Speciality Research Unit, Department of Zoology, Faculty of Science, Kasetsart University, Bangkok, 10900 Thailand; 3 Biodiversity Center, Kasetsart University, Bangkok, 10900, Thailand; 4 Department of Biological Science, Faculty of Science, Ubon Ratchathani University, Ubon Ratchathani 34190, Thailand; 5 School of Science, Walailak University, Nakhon Si Thammarat, 80161, Thailand; 6 Section of Research & Collections, North Carolina Museum of Natural Sciences, Raleigh, NC, USA

**Keywords:** Distribution, Gekkota, integrative taxonomy, ND2 gene, multivariate analysis

## Abstract

A new karst-dwelling bent-toed gecko of the *Cyrtodactylusintermedius* group is described from Khlong Hat District, Sa Kaeo Province, eastern Thailand, based on an integrative taxonomic analysis of genetic data and morphological characteristics. Phylogenetic analyses using the mitochondrial NADH dehydrogenase subunit 2 (ND2) gene revealed that topotypes of *C.intermedius* were sister to a clade containing *C.kulenensis* from Cambodia, an unnamed lineage from Sakaerat Biosphere Reserve in Nakhon Ratchasima Province, Thailand, and the Khlong Hat lineage described here as *Cyrtodactyluskhlonghatensis***sp. nov.** Multivariate analyses of morphometric and meristic characters showed that *C.khlonghatensis***sp. nov.** is morphologically distinct from all other species in the group by having the combination of SVL 76.5–82.8 mm in adult males and 88.5 mm in an adult female; eight supralabial and nine infralabial scales; 30–32 paravertebral tubercles; 20 or 21 longitudinal rows of dorsal tubercles; 43 or 44 ventral scales; seven or eight expanded subdigital lamellae on the 4^th^ toe; 12 unmodified subdigital lamellae on the 4^th^ toe; 19 or 20 total subdigital lamellae on the 4^th^ toe; 31 or 32 total number of enlarged femoral scales; enlarged femoral and precloacal scales continuous; 6–8 pore-bearing precloacal scales in males; three or four rows of enlarged post-precloacal scales; 1–3 postcloacal tubercles; proximal femoral scales less than one-half the size of distal femoral scales; absence of interdigital pocketing between digits of forefeet and hindfeet; and posterior border of the nuchal loop rounded. Uncorrected pairwise genetic divergences (*p*-distances) between the new species and other species of the *intermedius* group ranged from 4.73–22.55%. The discovery of this new species exclusively in isolated karst formations from the Thai-Cambodia border suggests that there may be further undiscovered *Cyrtodactylus* in unexplored karst landscapes along the border of eastern Thailand and western Cambodia.

## ﻿Introduction

The bent-toed gecko genus *Cyrtodactylus* Gray, 1827, is one of the most diverse among reptiles and the third-largest vertebrate genus globally (Grismer et al. 2021a), with 354 recognized species to date (Uetz et al. 2024). This genus exhibits a wide-ranging geographic distribution across various regions and is predominantly found in Southeast Asia, with their distribution extending from South Asia through the Indo-Australian Archipelago (Oliver et al. 2008, 2016; Wood et al. 2012; Luu et al. 2016; Agarwal et al. 2018; Neang et al. 2020; Chan et al. 2023; Grismer et al. 2015; 2021a, 2021b, 2022; Nugraha et al. 2023; Riyanto et al. 2022). *Cyrtodactylus* species have successfully adapted and evolved to occupy a variety of environments and ecological niches within this extensive range, including terrestrial, arboreal, cave-dwelling, and various substrate specialists (Ngo et al. 2008; Geissler et al. 2019; Grismer et al. 2020a, 2020b; Riyanto et al. 2022; Yodthong et al. 2022). In Thailand, 48 nominal species of *Cyrtodactylus* occur throughout the mainland and adjacent offshore islands (Uetz et al. 2024). Their presence in such diverse regions underscores their adaptability to thrive in a range of habitats and implies a complex evolutionary history for the genus (Chomdej et al. 2022; Grismer et al. 2018a, 2018b, 2020b, 2021b, 2023a; Termprayoon et al. 2021a, 2021b, 2023; Yodthong et al. 2022).

*Cyrtodactylusintermedius* (Smith, 1917) was originally described from Khao Sebab (= Namtok Phlio National Park), Chanthaburi Province, eastern Thailand. Additional populations were later reported from throughout eastern and southern Thailand, extending through the Cardamom Mountains of Cambodia and southward to southern Vietnam (Taylor, 1963; Stuart and Emmet 2006; Geissler et al. 2019; Murdoch et al. 2019; Grismer et al. 2020a, 2021b). *Cyrtodactylusintermedius* is now considered to represent a complex of species (Ngo et al. 2010; Murdoch et al. 2019; Grismer et al. 2015, 2020a, 2023a) known as the *C.intermedius* group (Grismer et al. 2021b). The group is monophyletic and comprises 13 recognized species (Murdoch et al. 2019; Grismer et al. 2020a, 2021b, 2023a; Uetz et al. 2024). These species include *C.auralensis* Murdoch, Grismer, Wood, Neang, Poyarkov, Tri, Nazarov, Aowphol, Pauwels, Nguyen & Grismer, 2019; *C.bokorensis* Murdoch, Grismer, Wood, Neang, Poyarkov, Tri, Nazarov, Aowphol, Pauwels, Nguyen & Grismer, 2019; *C.cardamomensis* Murdoch, Grismer, Wood, Neang, Poyarkov, Tri, Nazarov, Aowphol, Pauwels, Nguyen & Grismer, 2019; *C.disjunctus* Grismer, Pawangkhanant, Idiiatullina, Trofimets, Nazarov, Suwannapoom & Poyarkov, 2023; *C.hontreensis* Ngo, Grismer & Grismer, 2008; *C.intermedius* (Smith, 1917); *C.kohrongensis* Grismer, Onn, Oaks, Neang, Sokun, Murdoch, Stuart & Grismer, 2020; *C.kulenensis* Grismer, Geissler, Neang, Hartmann, Wagner & Poyarkov, 2021; *C.laangensis* Murdoch, Grismer, Wood, Neang, Poyarkov, Tri, Nazarov, Aowphol, Pauwels, Nguyen & Grismer, 2019; *C.phuquocensis* Ngo, Grismer & Grismer, 2010; *C.regicavernicolus* Chhin, Neang, Chan, Kong, Ou, In, Samorn, Sor, Lou, Sin, Chhim, Stuart & Grismer, 2024; *C.septimontium* Murdoch, Grismer, Wood, Neang, Poyarkov, Tri, Nazarov, Aowphol, Pauwels, Nguyen & Grismer, 2019; and *C.thylacodactylus* Murdoch, Grismer, Wood, Neang, Poyarkov, Tri, Nazarov, Aowphol, Pauwels, Nguyen & Grismer, 2019. Of these 13 species, only two species occur in Thailand, *C.disjunctus* (southern Thailand) and *C.intermedius* (eastern Thailand). Members of the *C.intermedius* group are highly adaptable to different habitats, including karst formations, granitic montane areas, sandstone, and other non-elevated terrestrial habitats (Grismer et al. 2020a, 2021b). This adaptability is likely due to their ecological versatility and ability to thrive in a variety of environmental settings (Murdoch et al. 2019; Grismer et al. 2023a). Other divergent mitochondrial lineages have been reported, suggesting that additional species diversity might exist within the *C.intermedius* group (Grismer et al. 2021a, 2021b, 2023a). One major hindrance to delimiting species in the *C.intermedius* group has been the lack of topotypic genetic material from the type locality of the nominate species *C.intermedius*.

During fieldwork from 2022–2023, we conducted surveys for *Cyrtodactylus* at Chanthaburi and Sa Kaeo Provinces in eastern Thailand. An integrative taxonomic approach, combining morphological characters, mitochondrial DNA analysis, and ecological data, was employed to compare the specimens to other members of the *C.intermedius* group and determine their taxonomic status. Additionally, samples were obtained from the type locality of *C.intermedius*. Herein, a distinct population from Khlong Hat District, Sa Kaeo Province is described as a new species.

## ﻿Materials and methods

### ﻿Sampling and specimen collection

**Figure 1.**
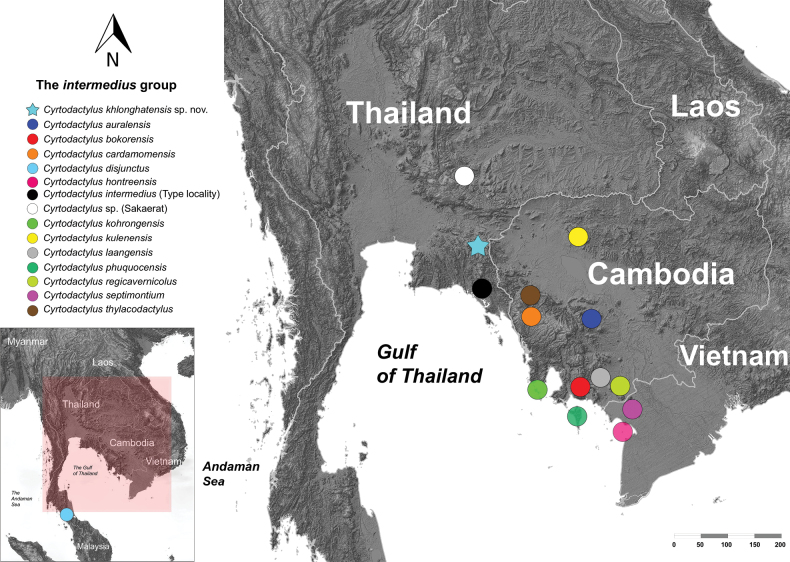
Map illustrating the distribution of the species of the *Cyrtodactylusintermedius* group using QGIS 3.34.8 (QGIS Development Team 2024). The elevation background data was derived from NASA LP DAAC (2013).

Field sampling was carried out through visual encounter surveys conducted both during the daytime (1000–1700 h) and at night (1900–2200 h) from July 2022 to February 2023 in two locations of eastern Thailand: (1) Khlong Hat District, Sa Kaeo Province and (2) Namtok Phlio National Park, Mueang Chanthaburi District, Chanthaburi Province (Fig. 1). Geographical coordinates and elevation for each locality were recorded using a Garmin GPSMAP 64s. Environmental factors (ambient temperature and relative humidity) were collected using a Kestrel 400 Weather Meter. Data on habitat, including microhabitat preferences, habitat use, and substrate type were also recorded for each specimen. Specimens were hand-collected and kept individually in bags for photographing prior to their euthanization. Specimens were humanely euthanized with tricaine methanesulfonate (MS-222) solution. The MS-222 solution was freshly prepared on the day of its use for euthanasia (Conroy et al. 2009; Simmons 2015; American Veterinary Medical Association 2020). Liver tissue was removed from each euthanized specimen, preserved in 95% ethanol, and stored at -20 °C for molecular study.

Voucher specimens were initially preserved in 10% formalin solution and subsequently transferred to 70% ethanol for morphological study and long-term storage. All specimens and tissue samples are deposited in the herpetological collection at the Zoological Museum of Kasetsart University, Bangkok, Thailand (**ZMKU**). Additional data were obtained from the original species descriptions of the *C.intermedius* group (Smith, 1917, 1935; Ngo et al. 2008; 2010; Murdoch et al. 2019; Grismer et al. 2020a, 2021b, 2023a; Chhin et al. 2024).

### ﻿Mitochondrial DNA analyses

Genomic DNA of the seven newly collected specimens (*C.intermedius* from the type locality, *n* = 4, and the Khlong Hat population, *n* = 3) was isolated from liver tissue samples using the Qiagen DNAeasy^TM^ Blood & Tissue Kit (Qiagen, Germany). A partial fragment of the mitochondrial NADH dehydrogenase subunit 2 (ND2) gene and its flanking tRNAs were amplified by polymerase chain reaction (PCR) under the following conditions: initial denaturation (95 °C, 2 min) followed by 31 cycles of a second denaturation (95 °C, 35 s), annealing (56 °C, 35 s), extension (72 °C, 35 s), and a final extension (72 °C, 10 min) using the light strand primer, L4437b (5’-AAGCAGTTGGGCCCATACC-3’; Macey et al. 1997) and the heavy strand primer, H5934 (5’ -AGRGTGCCAATGTCTTTGTGRTT-3’; Macey et al. 1997). All PCR products were purified using the QIAquick PCR Purification Kit (Qiagen Ltd., Hilden, Germany) and sequenced using the amplifying primers on an ABI 3730XL automatic sequencers (Applied Biosystems, CA, USA) with BigDye version 3 chemistry and the amplifying primers (Applied Biosystems, CA, USA). DNA sequences were edited and manually checked in Geneious Prime 2022.2.1 (Biomatters Ltd., Auckland, New Zealand). All newly generated sequences were deposited in GenBank under accession numbers PP444475–PP444481. All 42 sequences of *C.intermedius* group species and the five outgroups *C.oldhami* (Theobald, 1876), *C.trigroides* Bauer, Sumontha & Pauwels, 2003, *C.zebraicus* Taylor, 1962, *Dixoniussiamensis* (Boulenger, 1898), and *Hemidactylusfrenatus* Duméril & Bibron, 1836 were downloaded from GenBank (Suppl. material 1) following Ngo et al. (2010), Murdoch et al. (2019), Grismer et al. (2020a, 2021b), and Yodthong et al. (2022). The recently described species *C.regicavernicolus* was not included in the analyses but is closely related to *C.laangensis* (see Chhin et al. 2024). All downloaded sequences were aligned to the newly generated sequences using the MUSCLE plug-in as implemented in Geneious Prime 2022.2.1. The aligned dataset was partitioned by ND2 codon position and the flanking tRNAs.

Maximum Likelihood (ML) and Bayesian Inference (BI) analyses were used to estimate the phylogenetic relationships within the *C.intermedius* group. ModelFinder function within IQ-TREE (Kalyaanamoorthy et al. 2017) was used to select the best partitions for the ND2 gene and tRNAs for both ML and BI analyses. The selection was based on the Bayesian Information Criterion (BIC). For the ML analysis, TIM+F+G4 was identified as the best-fit model for codon partitions, and TN+F+G4 for the flanking tRNAs partitions. The ML analysis was conducted by the IQ-TREE webserver (Trifinopoulos et al. 2016) with 10,000 bootstrap pseudo-replicates employing the ultrafast bootstrap approximation algorithm (UFB; Minh et al. 2013; Hoang et al. 2018) to construct a final consensus ML phylogenetic tree. Nodes with ultrafast bootstrap supported values of 95 and above were considered strongly supported (Minh et al. 2013).

The BI analysis was conducted using MrBayes v. 3.2.7a on XSEDE (Ronquist et al. 2012) through the CIPRES Science Gateway (Miller et al. 2010). The BI analysis used default prior and GTR+I+Γ model of evolution for the codon partitions and flanking tRNAs. Two simultaneous runs were performed with four chains per run (three heated chains and one cold chain), using the default priors setting, a chain temperature set to 0.1, and 20 million generations sampled every 2,000 generations from the Markov Chain Monte Carlo (MCMC) chains. The first 25% of each run was discarded as burn-in using the “sumt” command. The stationary states of each parameter based on the standard deviation of split frequencies < 0.01 and the effective sample size (ESS) score above 200 for all parameters were assessed in Tracer v. 1.7.1 (Rambaut et al. 2018). The 50% majority-rule consensus of sampled tree from the post burn-in tree of the BI analysis and the most likely tree in the ML analysis were visualized and edited in FigTree v. 1.4.4 (Rambaut 2018). Nodes with Bayesian posterior probabilities support (BPP) of 0.95 and above were considered strongly supported (Huelsenbeck and Ronquist 2001; Wilcox et al. 2002). Uncorrected pairwise genetic divergences (*p*-distances) were estimated in MEGA11 (Tamura et al. 2021) using bootstrap method with 1,000 replications and the complete deletion option to remove missing data.

Voucher abbreviations are the School of Agriculture and Natural Resources, University of Phayao **(AUP)**, Aaron M. Bauer field series **(AMB)**, Chulalongkorn University Museum of Zoological Records, Bangkok, Thailand**(CUMZR)**, the Field Museum of Natural History, Chicago, Illinois, USA**(FMNH)**, Institut Royal des Sciences Naturelles de Belgique, Belgium**(IRSNB)**, the Institute of Tropical Biology Collection of Zoology in Ho Chi Minh City, Vietnam **(ITBCZ)**, La Sierra University Herpetological Collection**(LSUHC)**, the Zoological Research Museum Alexander Koenig, Bonn, Germany**(ZFMK)**, the Zoological Museum of Kasetsart University **(ZMKU)**, the corresponding Sabira S. Idiiatullina field number of the Zoological Museum of Moscow State University**(ZMMU ISS)** and the corresponding Nikolay A. Poyarkov field numbers of the Zoological Museum of Moscow State University**(ZMMU NAP)**.

### ﻿Morphological analyses

Coloration and patterns in life were assessed through digital images of individuals across all available age groups prior to preservation, taken by AR. Mensural, meristic, and qualitative characters were recorded by the first author on the left side of specimens for symmetrical traits using digital Mitutoyo CD-6″ ASX Digimatic Calipers to the nearest 0.1 mm under a Nikon SMZ 745 dissecting stereomicroscope. Only adult individuals, determined by the presence of secondary sexual characteristics such as the presence of large pore-bearing precloacal scales or hemipenial swelling in males, or visible eggs on the ventral side of the body in females, were included for morphological measurements and analyses. A total of 32 morphological characters (16 mensural characters and 16 meristic characters) were modified from previous studies of the *C.intermedius* group (Murdoch et al. 2019; Grismer et al. 2020a, 2021b).

Mensural measurements were as follows:

**SVL** snout to vent length, taken from tip of snout to the anterior margin of vent;

**TW** tail width, taken at the base of the tail immediately posterior to the postcloacal swelling;

**TL** tail length, taken from the vent to the tip of the tail, original or regenerated;

**FL** forearm length, taken on the dorsal surface from the posterior margin of the elbow while flexed 90° to the inflection of the flexed wrist;

**TBL** tibia length, taken on the ventral surface from the posterior surface of the knee while flexed 90° to the base of the heel;

**HL** head length, distance from the posterior margin of the retroarticular process of the lower jaw to the tip of the snout;

**HW** head width, measured at the angle of the jaws;

**HD** head depth, the maximum height of head from the occiput to the throat);

**AG** axilla to groin length, taken from the posterior margin of the forelimb at its insertion point on the body to the anterior margin of the hind limb at its insertion point on the body;

**ED** eye diameter, the maximum horizontal diameter of the eyeball;

**EE** eye-ear distance, measured from the anterior margin of the ear opening to the posterior edge of the eyeball;

**EL** ear length, taken from the greatest vertical distance of the ear opening;

**EN** eye to nostril distance, measured from the anterior most margin of the eyeball to the posterior margin of the external nares;

**ES** eye to snout distance, measured from the anterior margin of the eyeball to the tip of snout;

**IN** internarial distance, measured between the nares across the rostrum;

**IO** interorbital distance, measured between the anterior edges of the orbit.

Meristic characters were as follows:

**SL** the number of supralabial scales, counted from the largest scale immediately below the middle of the eyeball to the rostral scale;

**IL** the number of infralabial scales, counted from the mental to the termination of enlarged scales just after the upturn of the mouth;

**PVT** the number of paravertebral tubercles between limb insertions, counted in a straight line immediately left of the vertebral column;

**LRT** the number of longitudinal rows of body tubercles, counted transversely across the center of the dorsum from one ventrolateral fold to the other;

**VS** the number of longitudinal rows of ventral scales, counted transversely across the center of the abdomen from one ventrolateral fold to the other;

**4SLU** the number of small, unmodified subdigital lamellae distal to the digital inflection on the 4^th^ toe, counted from the digital inflection to the claw;

**4SLE** the number of expanded subdigital lamellae proximal to the digital inflection on the 4^th^ toe, counted from the base of the first phalanx where it contacts the body of the foot to the largest scale on the digital inflection;

**4SLT** the total number of subdigital lamellae beneath the 4^th^ toe;

**FS** The total number of enlarged femoral scales from each thigh combined as a single metric;

**PS** the number of enlarged precloacal scales;

**PP** the number of precloacal pores in males;

**PPS** the number of rows of post-precloacal scales on the midline between the enlarged precloacal scales and the vent;

**PCT** the number of postcloacal tubercles on either side of the base of the tail;

**BB** the number of dark body bands between limb insertions;

**LCB** the number of light caudal bands on the original tail;

**DCB** the number of dark caudal bands on the original tail.

Additional categorical characters examined were enlarged femoral and cloacal scales continuous or separated by a diastema at the base of the femora; proximal femoral scales were less than one-half the size of the distal femoral scales; and the presence or absence of a pocket in the skin webbing between the digits of the hind and forefeet. Color pattern characters examined were the nuchal loop being continuous from eye to eye or separated medially into paravertebral sections; the posterior border of the nuchal loop rounded or chevron-shaped to a point; the presence or absence of dark pigmented blotches on the top of the head; light-colored caudal bands encircling tail or not; regenerated tail bearing a pattern of dark spots or not. Morphological comparisons were based on examination of the original descriptions of species in the literature (Ngo et al. 2010; Murdoch et al. 2019; Grismer et al. 2015, 2020a, 2021b, 2023a).

Thirteen morphometric variables were size-adjusted for differences in ontogenetic composition by the allometric equation: X_adj_ = log[*X* ± β(SVL ± SVL_mean_)], where X_adj_ is the adjusted value of the morphometric variable; *X* is the unadjusted value of dependent variable; β = unstandardized regression coefficient for each species; SVL is snout to vent length; and SVL_mean_ is overall mean of SVL of each allometry species (Thorpe 1975, 1983; Turan 1999; Lleonart et al. 2000) using the R package “GroupStruct” (Chan and Grismer 2021) in the software R v.4.0.1 (R Core Team, 2020). Three morphological variables, including TL (tail length), TW (tail width), and EL (ear length), were excluded from the analyses due to differences in their conditions. Thirteen size-adjusted morphometric variables (SVL_adj_, FL_adj_, TBL_adj_, AG_adj_, HL_adj_, HW_adj_, HD_adj_, ED_adj_, EE_adj_, EN_adj_, ES_adj_, IN_adj_, and IO_adj_) were tested for normality using the Shapiro-Wilk test (*p* ≥ 0.05). Normality of data was confirmed for homogeneity of variances using Levene’s test (*p* ≥ 0.05) through the Paleontological statistics software (PAST version 4.11; Hammer et al. 2001).

Statistical analyses were performed to compare differences in morphological characteristics, body size, and shape within the *intermedius* group (*n* = 58), including populations from Khlong Hat samples (*n* = 4) and nine congener species: *C.auralensis* (*n* = 6), *C.bokorensis* (*n* = 7), *C.cardamomensis* (*n* = 6), *C.intermedius* (topotypes; *n* = 5), *C.kohrongensis* (*n* = 6), *C.kulenensis* (*n* = 9), *C.laangensis* (*n* = 5), *C.septimontium* (*n* = 7), *C.thylacodactylus* (*n* = 3) (Suppl. material 2). Due to lack of available measurements and small sample size, four species in the *intermedius* group (*C.disjunctus*, *C.hontreensis*, *C.phuquocensis* and *C.regicavernicolus*) were not included in the morphological analyses. Multivariate analyses employed 13 morphometric characters (SVL_adj_, FL_adj_, TBL_adj_, AG_adj_, HD_adj_, HL_adj_, HW_adj_, ED_adj_, EE_adj_, EN_adj_, ES_adj_, IN_adj_, and IO_adj_) and 10 meristic characters data (SL, IL, PVT, LRT, VS, 4SLU, 4SLE, 4SLT, FS, and PS). Femoral and precloacal pores were omitted from the multivariate analyses due to their presence only in males. Morphometric and meristic characters were concatenated into a single dataset and analyzed by principal component analysis (PCA) using the built in R functions: “prcomp” (R Core Team, 2020) and “ggplot2” (Wickham 2016) to find the best low-dimensional space character variation in data set and to reduce noise and the potential of overfitting. A discriminant analysis of principal components (DAPC) was performed using the “adegenet” package in R (Jombart 2008) to characterize clustering and distance separation in the morphospace of new groups, defined in the PCA, in comparison to nine congeners of the *intermedius* group. It was also used to generate linear combinations of centroids with the highest between-group variance (Jombart et al. 2010). Prior to plotting, dimension reduction in the DAPC involves preserving the initial set of principal components (PCs) that collectively explain approximately 90% of the variation within the dataset (Jombart and Collins 2015), a determination derived from a scree plot generated during the analysis. Maintaining an excessive number of PCs may introduce artificial structure into the data, whereas retaining too few run the risk of overlooking genuine structure (Cangelosi and Goriely 2007).

## ﻿Results

### ﻿Molecular analyses

**Figure 2.**
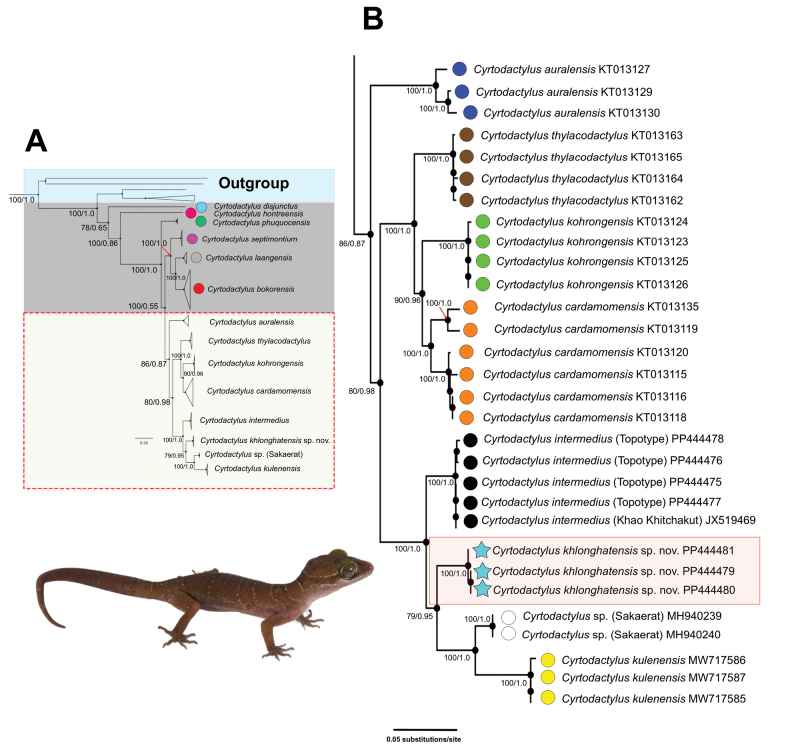
A best maximum likelihood topology illustrating the relationships of the *Cyrtodactylusintermedius* group and other related species based on 1,227 bp of the ND2 gene and flanking tRNAs **A** shown in full view **B** relevant clades of the *intermedius* group in close-up view. Nodal support values are ultrafast bootstrap values (UFB) from maximum likelihood analysis followed by posterior probabilities (BPP) of Bayesian analysis.

The total aligned dataset contained 1,227 characters of 49 individuals of the *C.intermedius* group and five individuals of the outgroup species (Fig. 2). The maximum likelihood value of the best ML tree was lnL = -26,799.981. The standard deviation of split frequencies was 0.002503 between the two simultaneous BI runs and the ESS values were ≥ 14,230 for all parameters. The results of ML and BI phylogenetic analyses recovered identical topologies (Fig. 2). The Khlong Hat samples represented a well-supported monophyletic lineage (100 UFB, 1.0 BPP) nested within the *C.intermedius* group (Fig. 2). The Khlong Hat population was strongly supported for BI (0.95 BPP) but not in ML (79 UFB) as the sister lineage to the clade containing *C.kulenensis* and *Cyrtodactylus* sp. from Sakaerat Biosphere Reserve, Nakhon Ratchasima Province (Fig. 2). The Khlong Hat population had uncorrected *p*-distances of 4.73–5.09% from *C.intermedius* (topotypes), 6.71–6.96% from *C.intermedius* (Khao Khitchakut), 5.82% from *Cyrtodactylus* sp. (Sakaerat) and 4.73–22.55% from other species in the *intermedius* group (Suppl. material 3). The within population uncorrected *p*-distances of the Khlong Hat population was 0.00%.

*Cyrtodactylusintermedius* samples from Namtok Phlio National Park (topotypes) and Khao Khitchakut, Chanthaburi Province, were recovered as a well-supported lineage (100 UFB, 1.0 BPP) and are the well-supported (100 UFB, 1.0 BPP) sister taxon to a clade comprised of *C.kulenensis*, *Cyrtodactylus* sp. from Sakaerat Biosphere Reserve and the Khlong Hat samples. *Cyrtodactylusintermedius* had uncorrected *p*-distance of 4.73–22.91% from other species in the *C.intermedius* group. The intraspecific uncorrected *p*-distances of *C.intermedius* was 0.00–1.87% (0.00–1.09% within the type locality; 1.75–1.87% between the type locality and Khao Khitchakut).

### ﻿Morphological analyses

**Figure 3.**
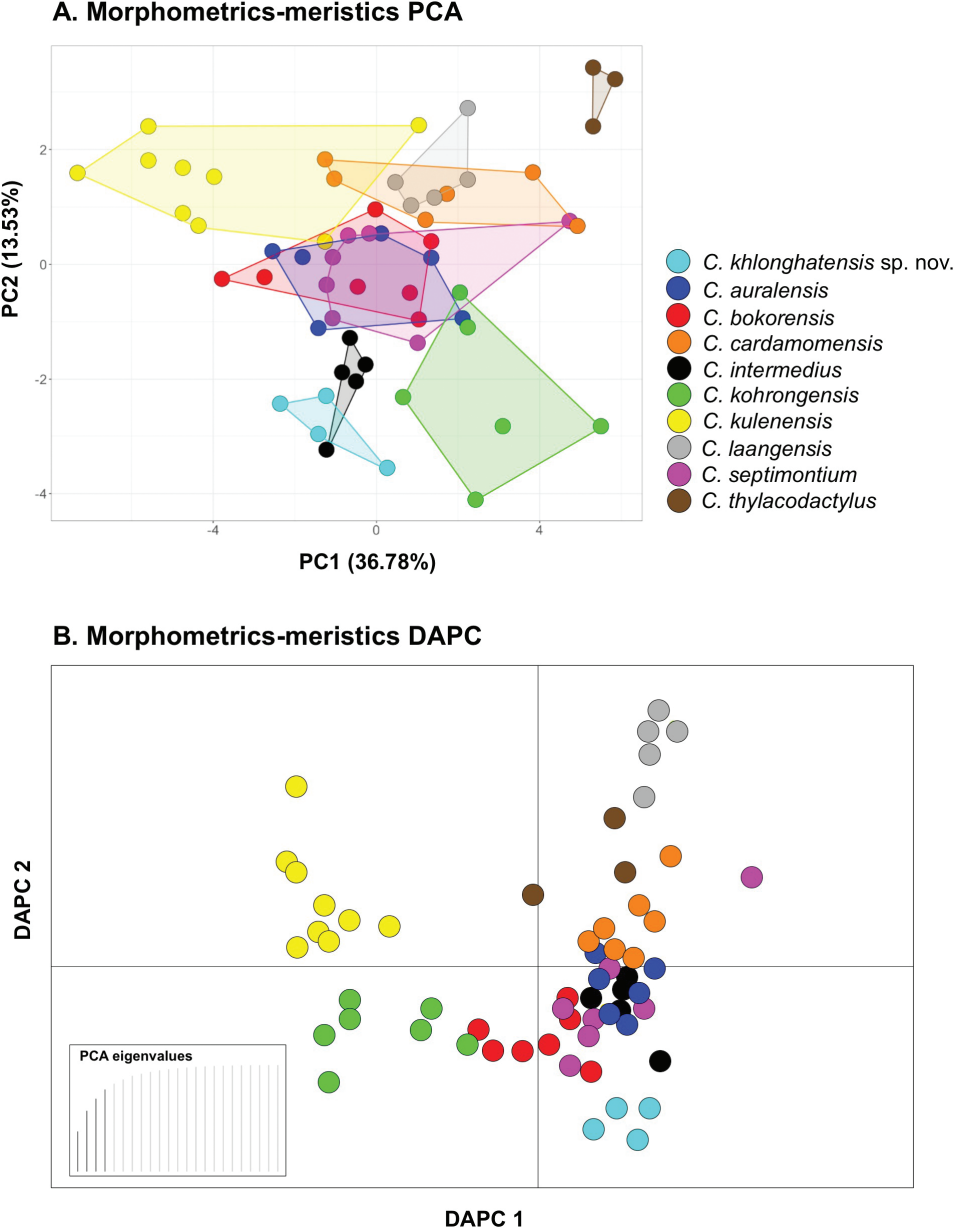
Multivariate analysis results of principal component analysis (PCA) and discriminant analysis of principal component (DAPC) of 23 morphological variables for ten species (*n* = 59 individuals) of the *intermedius* group **A**PCA scatterplot showing morphospatial differentiation among ten species in the *intermedius* group **B**DAPC plot based on the retention of 4 PC axes and discriminant eigenvalues showing morphospatial variation among ten species in the *intermedius* group.

Multivariate analyses using PCA and DAPC of Khlong Hat samples and nine species in the *C.intermedius* group revealed morphospatial differences along the ordination of the first two components and accounted for 50.31% of the variation (Fig. 3). The first six components of the PCA with eigenvalues > 1.0 accounted for 80.47% of the variation in the dataset (Table 1). PC1 explained for 36.78% of the variation and was heavily loaded with body size and head size (SVL_adj_, HL_adj_, HW_adj_). PC2 accounted for 13.53% of the variation and was heavily loaded on VS, 4SLE, 4SLU, and 4SLT. PC3–PC6 accounted for 11.76%, 7.09%, 6.50% and 4.81% of the variation, respectively and were heavily loaded on IO_adj_, SL, IL, 4SLE, 4SLU, LRT, FS, and PS (Table 1). The ordination of the first two components showed that the Khlong Hat samples clustered separately from all other species except *C.intermedius* (overlapped with one specimen). Factor loadings for each component of the morphometric and meristic characters data are provided in Table 1. The DAPC (76.89% of cumulative variance) showed strong separation of the Khlong Hat samples from all other species in the *C.intermedius* group (Fig. 3B).

**Table 1.** Summary of eigenvalues, standard deviation, percentage of variance, and factor loadings from the first six principal components (PC) of 13 size-adjusted morphometric and ten meristic characters of *Cyrtodactyluskhlonghatensis* sp. nov., and nine congeners of the *intermedius* group including *C.auralensis*, *C.bokorensis*, *C.cardamomensis*, *C.intermedius*, *C.kohrongensis*, *C.kulenensis*, *C.laangensis*, *C.septimontium*, and *C.thylacodactylus*. Values highlighted in bold represent those with the greatest contribution (factor loading ≥ 0.300) to the first six PCs with eigenvalue > 1.0. Measurement abbreviations are defined in the text.

	PC1	PC2	PC3	PC4	PC5	PC6
Eigenvalue	8.458	3.112	2.704	1.629	1.496	1.107
Standard deviation	2.908	1.764	1.644	1.277	1.223	1.052
% of variance	36.78	13.53	11.76	7.09	6.50	4.81
SVL_adj_	-**0.303**	0.055	-0.189	0.049	-0.125	0.050
FL_adj_	-0.280	0.134	-0.037	0.271	0.011	-0.011
TBL_adj_	-0.287	0.084	-0.006	0.278	0.009	-0.162
AG_adj_	-0.209	0.226	-0.249	0.187	-0.157	0.224
HL_adj_	-**0.300**	-0.129	-0.168	-0.021	-0.080	-0.078
HW_adj_	-**0.305**	-0.025	-0.161	-0.029	-0.099	0.079
HD_adj_	-0.284	0.119	0.149	-0.121	0.197	0.065
ED_adj_	-0.267	0.100	0.155	-0.023	-0.219	-0.127
EE_adj_	-0.262	-0.125	-0.160	-0.161	0.166	0.045
ES_adj_	-0.292	-0.113	0.063	-0.025	0.280	-0.036
EN_adj_	-0.281	-0.151	0.002	-0.077	0.213	-0.008
IN_adj_	-0.088	0.163	-0.173	0.284	0.287	-0.118
IO_adj_	-0.183	0.038	**0.355**	0.010	0.122	0.269
SL	-0.138	-0.265	0.183	-0.210	-0.207	-**0.370**
IL	-0.055	0.274	-0.046	-0.008	-**0.447**	-**0.537**
PVT	-0.205	0.170	0.181	-0.248	-0.243	0.159
LRT	-0.017	-0.103	-0.232	-**0.527**	-0.142	0.029
VS	-0.079	-**0.305**	-0.237	-0.104	-0.033	0.131
4SLE	-0.125	-**0.341**	**0.358**	-0.024	0.035	-0.107
4SLU	0.046	-**0.325**	-0.064	**0.424**	-**0.382**	0.223
4SLT	-0.058	-**0.446**	0.203	0.270	-0.223	0.090
FS	0.013	-0.201	-**0.481**	-0.100	-0.020	0.077
PS	-0.042	0.248	0.180	-0.161	-0.296	**0.505**

### ﻿Taxonomic hypotheses

The Khlong Hat population is clearly distinct from all other members of the *C.intermedius* group, as evidenced by the convergence of multiple analyses, including the phylogeny, multivariate analyses, and discrete diagnostic morphological characters (see “Comparison” below). Therefore, we hypothesize that the Khlong Hat population represents a distinct species that is described below as new.

### ﻿Systematics

#### ﻿ Cyrtodactylus khlonghatensis sp. nov.

Taxon classificationAnimaliaSquamataGekkonidae

3BDF08EB-BB2F-517E-974E-D4EAA64640E2

https://zoobank.org/595F31AB-E56F-436B-951A-633B3703EE40

**Figure 4.**
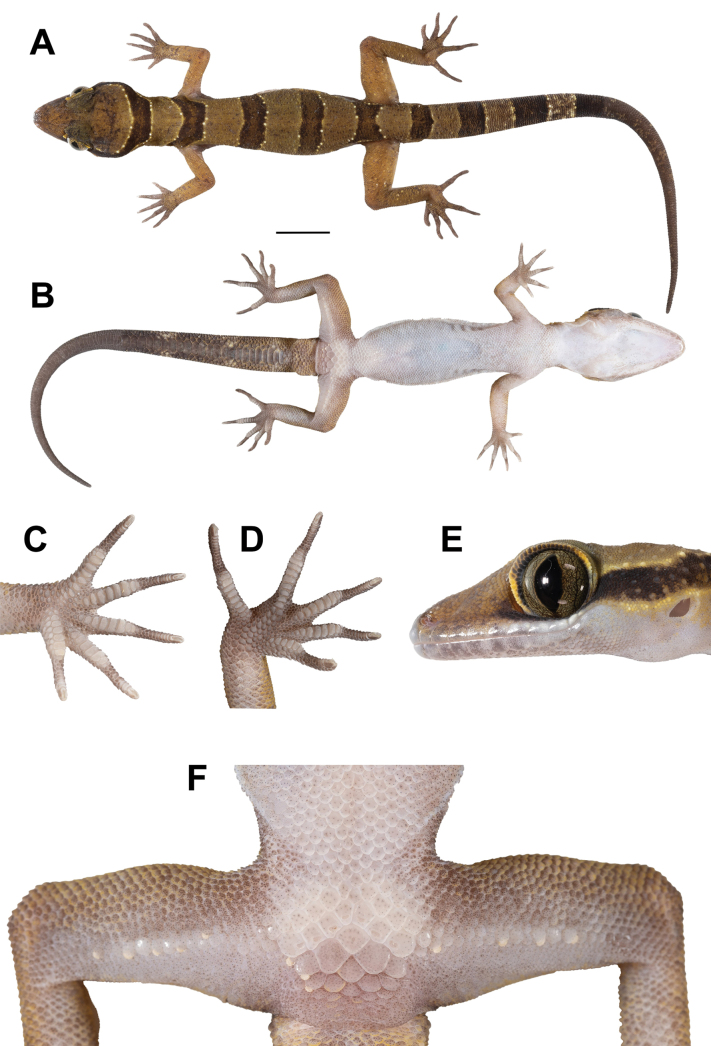
Adult male holotype of *Cyrtodactyluskhlonghatensis* sp. nov. (ZMKU R 01068) from Tham Phet Pho Thong, Khlong Hat Subdistrict, Khlong Hat District, Sa Kaeo Province, Thailand, prior to preservation **A** dorsal view **B** ventral view **C** palmar view of the right hand **D** plantar view of the right foot **E** lateral view of left side of head, and **F** precloacal region showing distribution of enlarged femeroprecloacal scales. Scale bar in dorsal and ventral views: 10 mm.

**Figure 5.**
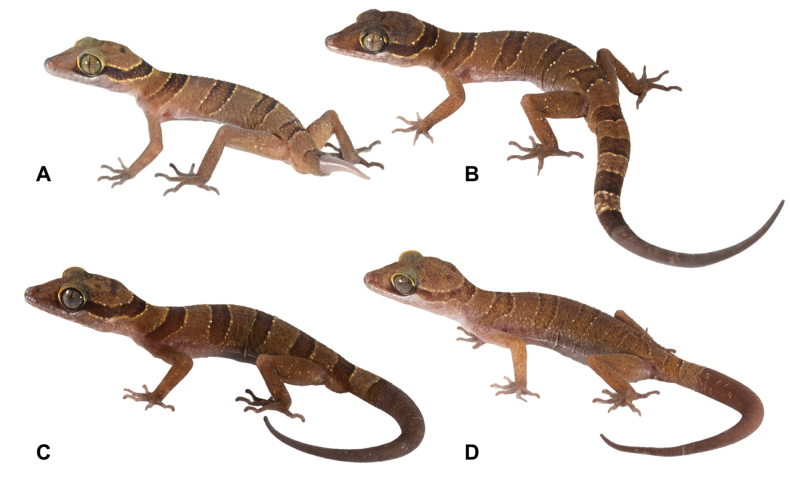
Variation in color pattern of *Cyrtodactyluskhlonghatensis* sp. nov. Tham Phet Pho Thong, Khlong Hat Subdistrict, Khlong Hat District, Sa Kaeo Province, Thailand, in life **A** adult male paratype (ZMKU R 01067) **B** adult male holotype (ZMKU R 01068) **C** adult male paratype (ZMKU R 01069), and **D** adult female paratype (ZMKU R 01070).

**Figure 6.**
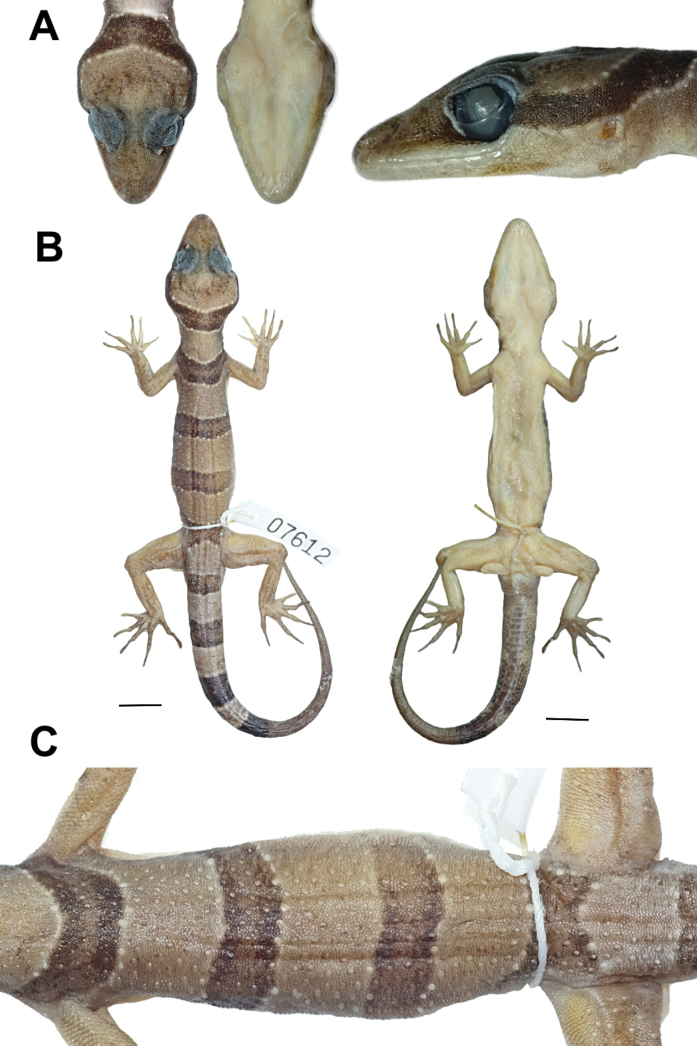
Adult male holotype of *Cyrtodactyluskhlonghatensis* sp. nov. (ZMKU R 01068; field number AA 07612) from Tham Phet Pho Thong, Khlong Hat Subdistrict, Khlong Hat District, Sa Kaeo Province, Thailand, in preservation **A** head dimensions showing dorsal, ventral, and lateral views **B** dorsal and ventral views **C** dorsal view of trunk. Scale bars in dorsal and ventral views: 10 mm.

Figs 4 , 5 , 6 Suggested common name: Khlong Hat Bent-toed Gecko

##### Type material.

***Holotype*** • ZMKU R 01068, adult male (Figs 4, 5B, 6) from Thailand, Sa Kaeo Province, Khlong Hat District, Khlong Hat Subdistrict, Tham (= cave) Phet Pho Thong (13°25.116'N, 102°19.690'E, 246 m elevation), collected on 28 July 2022 by Attapol Rujirawan. ***Paratypes*.** Five paratypes (three adults and two sub-adults) • Two adult males (ZMKU R 01067, ZMKU R 01069) and one adult female (ZMKU R 01070), same data as holotype • One sub-adult female (ZMKU R 01071), same data as holotype • One sub-adult male (ZMKU R 01072), same data as holotype, except from Khlong Kai Thuean Subdistrict, Tham Nam Khao Phra Siwa (13°19.258'N, 102°19.661'E, 178 m elevation), collected on 29 July 2022.

##### Diagnosis.

*Cyrtodactyluskhlonghatensis* sp. nov. can be distinguished from all other species of the *intermedius* group by having the following combination of characters: (1) SVL of 76.5–82.8 mm (mean 80.5 ± 3.5 mm, *n* = 3) in adult males and 88.5 mm in an adult female (*n* = 1); (2) eight supralabial and nine infralabial scales; (3) 30–32 paravertebral tubercles; (4) 20 or 21 longitudinal rows of dorsal tubercles; (5) 43 or 44 ventral scales; (6) seven or eight expanded subdigital lamellae on the 4^th^ toe; (7) 12 unmodified subdigital lamellae on the 4^th^ toe; (8) 19 or 20 total subdigital lamellae on the 4^th^ toe; (9) 31 or 32 total number of enlarged femoral scales; (10) enlarged femoral and precloacal scales continuous; (11) 6–8 pore-bearing precloacal scales in males; (12) three or four rows of enlarged post-precloacal scales; (13) 1–3 postcloacal tubercles; (14) proximal femoral scales < 1/2 the size of distal femoral scales; (15) absence of interdigital pocketing between digits of forefeet and hindfeet; and (16) posterior border of the nuchal loop rounded.

##### Description of holotype.

Adult male in good state of preservation with 82.8 mm SVL; head relatively moderate in length (HL/SVL 0.30), wide (HW/HL 0.64), slightly flattened (HD/HL 0.36), distinct from the neck, and triangular in dorsal profile; lores concave anteriorly, inflated posteriorly; frontal region flattened, prefrontal region concave; canthus rostralis rounded; snout rather elongate (ES/HL 0.40), rounded in the rostral region, eye to snout distance slightly greater than head depth; eye large (ED/HL 0.21), eyeball slightly protuberant, pupil vertical, the eye to ear distance greater than eye diameter; ear opening elliptical, obliquely oriented, moderate in size (EL/HL 0.07); rostral large, subrectangular, wider (3.3 mm) than high (1.8 mm), partially divided by a dorsal furrow, posteriorly bordered by left and right supranasals and smaller three internasal scales, laterodorsally bordered by nostril opening and 1^st^ supralabial; external nares anteriorly bordered by rostral, dorsally by large supranasal, posteriorly by two small postnasals, ventrally bordered by 1^st^ supralabial; 8L/8R subrectangular supralabials extending to below the center of the eye, 10L/10R to the posterior margin of the eyeball, subrectangular anteriorly, elliptical shape posteriorly; 2^nd^ to 6^th^ supralabials slightly larger than 1^st^ suprabial; 6L/6R infralabials extending to below center of the eye, 9L/9R to below the posterior margin of the eyeball, larger than supralabials, tapering smoothly posteriorly; scales of frontonasal, prefrontal and lores small, domed, relatively raise, slightly larger than granular scales on top of head and occiput; scales of occiput and top of head intermixed with scattered, distinct, enlarged tubercles, more prominent tubercles between occiput and ear opening; dorsal supraciliaries smooth, not elongate; mental large, triangular, 3.2 mm in width, 2.4 mm in length, laterally bordered by 1^st^ infralabial and posteriorly by large, left and right trapezoidal postmentals which contact medially for 50% of their length posterior to mental; one row of slightly enlarged, elongate sub-labials extending posteriorly to 7^th^ infralabials for both side; and gular and throat scales small, granular, grading posteriorly into larger, smooth, flat, imbricate, pectoral and ventral scales.

Body slender, relatively short (AG/SVL 0.41), with poorly-defined ventrolateral folds posteriorly; dorsal scales small, homogenous, granular, interspersed with relatively large, conical, semi-regularly arranged, slightly prominent trihedral keeled tubercles; tubercles extending from occipital region onto base of tail but end at regenerated tail, smaller at the anterior portion of body and increasing in size posteriorly; tubercles on occiput, nape and upper body at the level above shoulder smaller, subconical; mid-dorsally, on the posterior section of the body and tail larger, more dense, slightly more prominently keeled, semi-regularly arranged; approximately 21 longitudinal rows of dorsal tubercles between ventrolateral body folds at midbody; 32 paravertebral tubercles; 44 longitudinal rows of flat, imbricate smooth ventral scales between ventrolateral body fold much larger than dorsal scales; one row of 16L/15R enlarged femoral scales continuous with enlarged precloacal scales, enlarged femoral scales extending along 2/3 of the femora; proximal femoral scales < 1/2 size of distal femoral scales; femoral pores absent; seven enlarged, pore-bearing precloacal scales, smooth, approximately twice the size of femoral scales; precloacal groove or depression absent; three rows of enlarged post-precloacal scales.

Forelimbs rather slender, relatively short (FL/SVL 0.14); granular scales on forearm slightly larger than those on body, interspersed with enlarged, subconical smooth tubercles; dorsal scales of wrist and palm slightly rounded, flat, smooth, imbricate, slightly raise; ventral scales of palm flat, weakly rounded, smaller than those on body, slightly raised; 18L/18R total subdigital lamellae on 4^th^ finger; 7L/7R proximal subdigital lamellae rectangular with rounded, wide, transversely expanded proximal to joint inflection on 4^th^ finger, 11L/11R unmodified lamellae distal to inflection, gradually more expanded near the claw; digits narrower distal to inflections; interdigital pocketing absent on the forefeet; claws well-developed, relatively short, claw base sheathed by a dorsal and ventral scales; hindlimbs more robust than forelimbs, moderate in length (TBL/SVL 0.17); dorsal scales slightly rounded, granular, subconical, interspersed with enlarged subconical, smooth tubercles, and anteriorly by flat, slightly larger scales; ventral scales of femora flat, imbricate, smooth, larger than dorsals; ventral scales of tibia and subtibia flat, smooth, imbricate; 20L/20R total subdigital lamellae on 4^th^ toe, 8L/8R proximal subdigital lamellae, rectangular with rounded, wide, transversely expanded proximal to joint inflection on 4^th^ toe, 12L/12R unmodified lamellae distal to inflection, gradually more expanded near the claw; digits narrower distal to inflections; interdigital pocketing absent on the hindfeet; claws well-developed, short, claw base sheathed by a dorsal and ventral scales.

Tail regenerated, 100.5 mm in length, longer than SVL (TL/SVL 1.21), moderate in proportions, cylindrical, segmented, wide anteriorly, 7.7 mm in width at the base, tapering to a point, becoming slender toward the tip; dorsal scales of the original portion of tail small, flat, squared; dorsal scales of tail base granular, rounded, regenerated portion covered by small, smooth subcircular scales, grading posteriorly into larger, flatter; trihedral keeled tubercles forming paravertebral rows on tail base extending to posterior margin of 1/2 of tail; subcaudal scale rows enlarge, smooth; median row of transversely expanded subcaudal scales present, significantly larger than dorsal caudal scales; well-defined narrow ventrolateral subcaudal furrow present; tail base bearing hemipenial swellings; 3L/3R smooth, conical, flat, imbricate postcloacal tubercles on either side of hemipenial swellings; and postcloacal tubercles approximately equal in size.

##### Coloration of holotype in life.

(Figs 4, 5B). Dorsal ground color of head, body, and limbs light-brown; indistinct dark-brown markings on top of head; superciliary scales pale yellow anteriorly and posteriorly; iris brown with dark brown vermiculations; rostral and loreal regions dark brown; rostral, mental, supralabial and infralabial scales creamy-white with scattered dark brown pigment; dark brown nuchal loop with rounded posterior border extends from posterior margin of orbit to posterior margin of the other orbit; nuchal loop edged with thin, pale lines and creamy white tubercles; four similar dark brown body bands, edged in creamy white tubercles with slightly paler centers occur between limb insertion; first body band terminates at shoulders near anterior margin of forelimb insertion; second and third body bands terminate at dorsal to ventrolateral fold on flanks; fourth body band terminates at anterior margin of hindlimb insertion; limbs lighter brown; dorsal portion of forelimbs bearing scattered dark brown markings; dorsal portion of hindlimbs bearing pale yellow spots; four wide dark brown caudal bands encircling the original tail edged in creamy white tubercles; three wide pale caudal bands brown encircling tail; regenerated tail, uniformly brown with small, scattered creamy white markings dorsally; regenerated tail extending from posterior margin of 4^th^ dark caudal band.

Ventral surfaces of head, body, and limbs dull white to beige, stippled; ventral surfaces of fingers and toes with dark pigmentation; subdigital lamellae on fingers and toes off-white; palmar surface dark brown; hemipenial swelling dark brown with scattered pale yellow; subcaudal region darkened with fine mottling anteriorly.

##### Coloration in preservative.

(Fig. 6). Overall color pattern of head, body, limbs, flanks, and tail remains similar to that observed in life; dorsal ground color became pale brown in hue; all creamy white tubercles and scales on both dorsal and ventral surfaces faded to an off-white; dark body bands and dark caudal bands appear lighter than observed in life; entire ventral surfaces changed to greyish white with small, refined dark mottling; regenerated tail turned pale brown.

##### Variation.

All paratypes closely resemble the holotype in coloration (Fig. 5). Morphometric, meristic and color pattern characters of the type series of *C.khlonghatensis* sp. nov. are presented in Tables 2, 3. ZMKU R 01067 (adult male), ZMKU R 01069 (adult male), ZMKU R 01070 (adult female), ZMKU R 01071 (subadult female), and ZMKU R 01072 (subadult male) bear dark brown blotches on the top of the head. The adult female (ZMKU R 01070) exhibits a pale-colored nuchal loop, body, and caudal bands edged with creamy white tubercles. All paratypes have regenerated tails, except for two subadult specimens (ZMKU R 01071–01072), which retain their original tails with a caudal band encircling the tail edge. Posterior portion of tail in juveniles (not collected) white.

**Table 2.** Descriptive measurements of the type series (adult) of *Cyrtodactyluskhlonghatensis* sp. nov. in millimeters. Abbreviations are defined in Materials and methods. Key: *n* = number.

Characters	Holotype male	Holotype and paratypes males		Paratype females
	*n* = 1	*n* = 3		*n* = 1
		Min–Max	Mean ± SD	
SVL	82.8	76.5–82.8	80.5 ± 3.5	88.5
AG	33.8	33.7–33.9	33.8 ± 0.1	38.4
ED	5.2	5.1–5.3	5.2 ± 0.1	5.7
EE	7.1	6.9–7.2	7.1 ± 0.1	7.4
EL	1.7	1.6–1.8	1.7 ± 0.1	1.7
EN	7.7	7.4–7.9	7.7 ±0.2	7.7
ES	9.8	9.6–9.9	9.7 ± 0.2	9.5
FL	11.7	11.6–11.7	11.7 ± 0.1	12.0
HD	9.0	8.3–9.0	8.8 ± 0.4	9.2
HL	24.7	23.4–24.7	24.1 ± 0.6	25.4
HW	15.9	15.2–15.9	15.6 ± 0.4	16.1
IN	2.4	2.2–2.4	2.3 ± 0.1	2.2
IO	3.5	3.2–3.5	3.4 ± 0.2	3.2
TBL	14.1	13.9–14.2	14.1 ± 0.2	14.4
TL (original)	–	–	–	–
TL (regenerated)	100.5	20.8–100.5	69.2 ± 42.6	86.5
TW	7.7	7.2–7.7	7.4 ± 0.2	7.4

**Table 3.** Morphological data for the type series of *Cyrtodactyluskhlonghatensis* sp. nov. Abbreviations are defined in Materials and methods. Key: re = regenerated tail; L = left; R = right; NA = not applicable.

Characters	ZMKU R 01068	ZMKU R 01067	ZMKU R 01069	ZMKU R 01070	ZMKU R 01071	ZMKU R 01072
Type	**Holotype**	**Paratype**	**Paratype**	**Paratype**	**Paratype**	**Paratype**
Sex	Male	Male	Male	Female	Subadult-female	Subadult- male
SVL	82.8	76.5	82.2	88.5	65.9	64.2
TL	100.5re	20.8re	86.3re	86.5re	85.5	80.1
TW	7.7	7.2	7.3	7.4	5.2	6.1
FL	11.7	11.6	11.6	12.0	9.3	9.4
TBL	14.1	13.9	14.2	14.4	10.4	10.9
AG	33.8	33.7	33.9	38.4	27.3	29.0
HL	24.7	23.4	24.2	25.4	19.2	18.9
HW	15.9	15.2	15.8	16.1	11.9	12.7
HD	9.0	8.3	8.9	9.2	6.9	7.1
ED	5.2	5.1	5.3	5.7	3.9	4.2
EE	7.1	6.9	7.2	7.4	5.8	5.6
ES	9.8	9.6	9.9	9.5	7.5	7.4
EN	7.7	7.4	7.9	7.7	5.7	5.5
EL	1.7	1.6	1.8	1.7	1.4	1.1
IN	2.4	2.2	2.3	2.2	1.9	1.9
IO	3.5	3.2	3.4	3.2	2.7	2.7
supralabials	8L/8R	8L/8R	8L/8R	8L/8R	8L/8R	8L/8R
infralabials	9L/9R	9L/9R	9L/9R	9L/9R	9L/9R	9L/9R
paravertebral tubercles	32	31	31	30	30	31
longitudinal rows of tubercles	21	21	20	21	20	20
ventral scales	44	44	44	43	43	43
expanded subdigital lamellae on 4^th^ toe	8	8	7	8	8	8
unmodified subdigital lamellae on 4^th^ toe	12	12	12	12	12	12
total subdigital lamellae on 4^th^ toe	20	20	19	20	20	20
sum of enlarged femoral scales	31 (16L/15R)	32 (16L/16R)	32 (16L/16R)	32 (16L/16R)	32 (16L/16R)	32 (16L/16R)
precloacal scales	7	6	8	8	8	7
precloacal pores	7	6	8	7 pits	8 pits	7
post-precloacal scales rows	3	4	3	4	4	3
postcloacal tubercles	3L/3R	2L/2R	3L/3R	2L/2R	1L/1R	2L/3R
body bands	4	4	4	4	4	4
femoral and precloacal scales continuous (yes or no)	yes	yes	yes	yes	yes	yes
proximal femoral scales < 1/2 size of distal femorals	yes	yes	yes	yes	yes	yes
pocketing between digits of hindfeet	no	no	no	no	no	no
pocketing between digits of forefeet	no	no	no	no	no	no
dark pigmented blotches on top of the head	no	yes	yes	yes	yes	yes
posterior border of the nuchal loop rounded or pointed	rounded	rounded	rounded	rounded	rounded	rounded
no. of dark caudal bands	NA	NA	NA	NA	10	10
no. of light caudal bands	NA	NA	NA	NA	9	9
dark caudal bands wider than light caudal bands	NA	NA	NA	NA	yes	yes

##### Distribution.

*Cyrtodactyluskhlonghatensis* sp. nov. is currently known from only two localities: (1) Tham Phet Pho Thong (type locality) in Khlong Hat District, Sa Kaeo Province, Thailand; and (2) Tham Nam Khao Phra Siwa, Khlong Kai Thuean Subdistrict, Khlong Hat District, Sa Kaeo Province, Thailand, approximately 10 km from the type locality.

##### Natural history.

**Figure 7.**
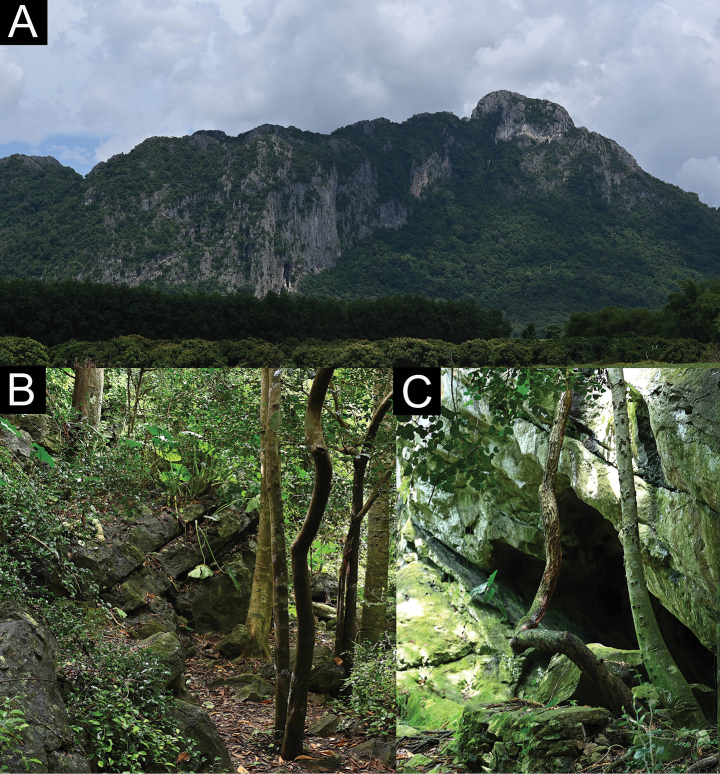
Habitats of *Cyrtodactyluskhlonghatensis* sp. nov. at the type locality of Tham Phet Pho Thong, Khlong Hat Subdistrict, Khlong Hat District, Sa Kaeo Province, Thailand **A** the isolated karstic mountain surrounded by karstic outcrops with dry deciduous forest **B** karstic trail **C** karst boulders.

The type locality is an isolated karstic formation mountain surrounded by karstic outcrops in dry deciduous forest at an elevation of 246 m. The type series of *C.khlonghatensis* sp. nov. was found during both day (1400–1530 h) and night (1900–2000 h) in various microhabitats of the Tham Phet Pho Thong karstic area (Fig. 7), including karstic boulders, karstic wall, cracks, and crevices; shrubs; vines and other vegetations. The male holotype was found at night (1950 h), perched on a dry vine near a karstic wall, approximately 20 cm above the ground. The male paratype (ZMKU R 01067) was found during the day on a karstic wall in a cave, approximately 5 m from the entrance, with air temperatures of 26.3 °C and a relative humidity of 93.3%. Another male paratype (ZMKU R 01069) was found at night on a karstic wall in a cave. The female paratype (ZMKU R 01070) was found perched on a dry log along a trail in a karstic habitat. A subadult male (ZMKU R 01071) was found perched upside down on a shrub, approximately 50 cm above ground level. At Tham Nam Khao Phra Siwa, a subadult female (ZMKU R 01072) was found on crevices of a karstic wall near a cave entrance, approximately 50 cm above the ground. *Cyrtodactyluskhlonghatensis* sp. nov. is likely a nocturnal species that inhibits karstic environments. During the day, individuals were found to be inactive in shaded areas with cracks, while at night, they were active both on the karstic terrain and in vegetation. In this survey, the smaller nocturnal gekkonid *Gehyramutilata* (Wiegmann, 1834) was found in sympatry on karstic boulders, karstic outcrops and vegetations such as tree trunks and dry shrubs.

##### Comparisons.

(Suppl. materials 3, 4). *Cyrtodactyluskhlonghatensis* sp. nov. is differentiated from 13 recognized species of the *intermedius* group by having a unique combination of morphological characteristics and uncorrected pairwise sequence divergences of mtDNA (ND2) of 4.73–22.55%.

*Cyrtodactyluskhlonghatensis* sp. nov. is distinguished from *C.auralensis* by having a larger maximum SVL of 88.5 mm (vs 84.3 mm); 20 or 21 longitudinal rows of body tubercles (vs 17 or 18 rows); and 31 or 32 total number of enlarged femoral scales (vs 23–28 scales).

*Cyrtodactyluskhlonghatensis* sp. nov. is distinguished from *C.bokorensis* by having a smaller maximum SVL of 88.5 mm (vs 93.0 mm); 31 or 32 total number of enlarged femoral scales (vs 26–30 scales); and posterior border of the nuchal loop rounded (vs pointed).

*Cyrtodactyluskhlonghatensis* sp. nov. is distinguished from *C.cardamomensis* by having a larger maximum SVL of 88.5 mm (vs 84.1 mm); 7 or 8 expanded subdigital lamellae proximal to the digital inflection on the 4^th^ toe (vs 5 or 6 lamellae); 31 or 32 total number of enlarged femoral scales (vs 23–28 scales); 6–8 precloacal pores (vs 9 or 10 pores); proximal femoral scales < 1/2 size of distal femoral scales present (vs absent); and dark pigmented blotches on top of the head varies (vs absent).

*Cyrtodactyluskhlonghatensis* sp. nov. is distinguished from *C.disjunctus* by having a larger maximum SVL of 88.5 mm (vs 66.7 mm); 8 supralabial scales (vs 12 scales); 9 infralabial scales (vs 11 scales); 30–32 paravertebral tubercles between limb insertions (vs 41 tubercles); 20 or 21 longitudinal rows of body tubercles (vs 11 rows); 43 or 44 longitudinal rows of ventral scales (vs 36 rows); 12 unmodified subdigital lamellae distal to the digital inflection on the 4^th^ toe (vs 9 lamellae); 19 or 20 total number of subdigital lamellae beneath the 4^th^ toe (vs 17 lamellae); 31 or 32 total number of enlarged femoral scales (vs 21 scales); 6–8 precloacal scales (vs 10 scales); 6–8 precloacal pores (vs 9 pits); 3 or 4 rows of post-precloacal scales (vs 1 row); enlarged femoral and precloacal scales continuous (vs discontinuous); 4 body bands (vs 3 bands); and dark pigmented blotches on top of the head varies (vs absent).

*Cyrtodactyluskhlonghatensis* sp. nov. is distinguished from *C.hontreensis* by having 8 supralabial scales (vs 11–13 scales); 30–32 paravertebral tubercles between limb insertions (vs 20–24 tubercles); 20 or 21 longitudinal rows of body tubercles (vs 14 rows); 43 or 44 longitudinal rows of ventral scales (vs 40–42 rows); 31or 32 total number of enlarged femoral scales (vs 4–9 scales); enlarged femoral and precloacal scales continuous (vs discontinuous); and 4 body bands (vs 3 bands).

*Cyrtodactyluskhlonghatensis* sp. nov. is distinguished from *C.intermedius* by having 31 or 32 total number of enlarged femoral scales (vs 23–26 scales); and dark pigmented blotches on top of the head varies (vs absent).

*Cyrtodactyluskhlonghatensis* sp. nov. is distinguished from *C.kohrongensis* by having a larger maximum SVL of 88.5 mm (vs 76.1 mm); 43 or 44 longitudinal rows of ventral scales (vs 38–42 rows); 31 or 32 total number of enlarged femoral scales (vs 14–26 scales); enlarged femoral and precloacal scales continuous (vs discontinuous); and dark pigmented blotches on top of the head varies (vs absent).

*Cyrtodactyluskhlonghatensis* sp. nov. is distinguished from *C.kulenensis* by having 30–32 paravertebral tubercles between limb insertions (vs 33–38 tubercles); 20 or 21 longitudinal rows of body tubercles (vs 17–19 rows); 31 or 32 total number of enlarged femoral scales (vs 10–21 scales); 6–8 precloacal scales (vs 9 or 10 scales); and dark pigmented blotches on top of the head varies (vs absent).

*Cyrtodactyluskhlonghatensis* sp. nov. is distinguished from *C.laangensis* by having a larger maximum SVL of 88.5 mm (vs 82.2 mm); 9 infralabial scales (vs 10–11 scales); 20 or 21 longitudinal rows of body tubercles (vs 17 or 18 rows); 43 or 44 longitudinal rows of ventral scales (vs 37–40 rows); and 31 or 32 total number of enlarged femoral scales (vs 0–16 scales).

*Cyrtodactyluskhlonghatensis* sp. nov. is distinguished from *C.phuquocensis* by having a larger maximum SVL of 88.5 mm (vs 85.8 mm); 8 supralabial scales (vs 9–13 scales); 20 or 21 longitudinal rows of body tubercles (vs 16–18 rows); 7 or 8 expanded subdigital lamellae proximal to the digital inflection on the 4^th^ toe (vs 5 or 6 lamellae); 31 or 32 total number of enlarged femoral scales (vs 21–28 scales); and dark pigmented blotches on top of the head varies (vs absent).

*Cyrtodactyluskhlonghatensis* sp. nov. is distinguished from *C.regicavernicolus* by having a larger maximum SVL of 88.5 mm (vs 80.7 mm); 20 or 21 longitudinal rows of body tubercles (vs 15–18 rows); 31 or 32 total number of enlarged femoral scales (vs 8–23 scales); and enlarged femoral and precloacal scales continuous (vs discontinuous).

*Cyrtodactyluskhlonghatensis* sp. nov. is distinguished from *C.septimontium* by having a smaller maximum SVL of 88.5 mm (vs 90.4 mm); 20 or 21 longitudinal rows of body tubercles (vs 16–19 rows); 43 or 44 longitudinal rows of ventral scales (vs 38–42 rows); and 31 or 32 total number of enlarged femoral scales (vs 24–28 scales)

*Cyrtodactyluskhlonghatensis* sp. nov. is distinguished from *C.thylacodactylus* by having a larger maximum SVL of 88.5 mm (vs 74.6 mm); 8 supralabial scales (vs 7 scales); 43 or 44 longitudinal rows of ventral scales (vs 36–42 rows); 7 or 8 expanded subdigital lamellae proximal to the digital inflection on the 4^th^ toe (vs 5 or 6 lamellae); 19 or 20 total number of subdigital lamellae beneath the 4^th^ toe (vs 15–18 lamellae); 31 or 32 total number of enlarged femoral scales (vs 17–22 scales); proximal femoral scales < 1/2 size of distal femoral scales present (vs absent); interdigital pocketing between digits of forefeet and hindfeet absent (vs present); and dark pigmented blotches on top of the head varies (vs absent).

##### Etymology.

The specific epithet *khlonghatensis* is named after the type locality of Khlong Hat Subdistrict, Khlong Hat District, Sa Kaeo Province, Thailand.

## ﻿Discussion

In recent decades, there has been a notable increase in research focusing on the taxonomy and systematics of *Cyrtodactylus*, especially in Southeast Asia (Welton et al. 2010; Johnson et al. 2012; Oliver et al. 2014; Grismer et al. 2020a, 2021a, 2021b, 2022, 2023a, 2023b; Chan et al. 2023; Ngo et al. 2022; Termprayoon et al. 2023; Uetz et al. 2024). Grismer et al. (2015) first noted that *C.intermedius* represented a species complex with ecomorphologically diverse characteristics. An integrative taxonomic approach, combining morphological, molecular data, and ecology, has played a crucial role in unveiling the hidden diversity within the *intermedius* group (Murdoch et al. 2019; Grismer et al. 2020a, 2021b, 2023a).

The discovery of *Cyrtodactyluskhlonghatensis* sp. nov. further highlights the remarkable endemism of gekkonids in the isolated hilly karstic regions of the Indo-Burma Hotspot (e.g., Grismer et al. 2014, 2018b; Murdoch et al. 2019; Rujirawan et al. 2019; Nguyen et al. 2020; Luu et al. 2023), while also demonstrating the adaptability of habitat preferences within the *C.intermedius* group. The discovery of this new species increases the total number of species in the *C.intermedius* group to 14, of which three occur in Thailand. These finding also suggest that additional members of the *C.intermedius* group may exist in Thailand, where vast unexplored karst landscapes remain. Further surveys are warranted to delineate the geographic distribution of *C.intermedius* in eastern and northeastern Thailand.

This study provides the first genetic data for *C.intermedius* from its type locality at Khao Sebab (= Namtok Phlio National Park, Mueang Chanthaburi District), Chanthaburi Province. We identified an error in the reported sampling locality of *C.intermedius* (LSUHC 9513) that was incorrectly listed as “Khao Soi Dao, Chanthaburi, Thailand” (see Murdoch et al. 2019: table 1), rectified here to the sampling locality of “Khao Khitchakut, Chanthaburi Province, Thailand” (Suppl. material 1). Our results support the hypotheses of Murdoch et al. (2019) that *C.intermedius* from Khao Khitchakut, located 30 km north of the type locality, is conspecific with true *C.intermedius*. This confirmation is also based on morphological comparisons between syntypes (UMMZ 78687, MCZ R 39040, and FMNH 215981; see Murdoch et al. 2019), the newly collected topotypic specimens from this study (ZMKU R 01037–01038 and ZMKU R 01044–01045), and the Khao Khitchakut population (LSUHC 9513; see Murdoch et al. 2019). The phylogenetic position of *C.intermedius* from the type locality was recovered as the sister species to a clade composed of three species: *C.khlonghatensis* sp. nov. from Khlong Hat District, Sa Kaeo Province, *Cyrtodactylus* sp. from Sakaerat Biosphere Reserve, Wang Nam Khiao District, Nakhon Ratchasima Province, and *C.kulenensis* from Phnom Kulen National Park, Phnom Kbal, Benteay Srei District, Siem Reap Province, Cambodia. The eastern-southern division of the *C.intermedius* group shown here is concordant with previous studies (Murdoch et al. 2019; Grismer et al. 2015, 2020a, 2021b, 2023a).

Thailand’s complex geological history is evident in the abundance of limestone and granite formations found in the eastern and northeastern regions (Day and Urich 2000; Morley et al. 2011). These karstic regions and granitic outcrops are revealing a rich diversity of reptiles, particularly species with limited ranges (Bauer and Das 1998; Bauer et al. 2002; Wood et al. 2010; Murdoch et al. 2019; Vogel and Patrick 2019; Grismer et al. 2019). To enhance our understanding of the taxonomy, ecology, distribution, biogeography, and conservation of *C.intermedius* group in eastern and northeastern Thailand, further research and additional field surveys in unexplored regions are imperative.

## Supplementary Material

XML Treatment for Cyrtodactylus khlonghatensis

## ﻿Additional information

### Author ORCIDs

Natee Ampai https://orcid.org/0000-0001-8562-299X

Attapol Rujirawan https://orcid.org/0000-0001-9179-6910

Siriporn Yodthong https://orcid.org/0000-0001-6577-5358

Korkhwan Termprayoon https://orcid.org/0000-0003-1903-3040

Bryan L. Stuart https://orcid.org/0000-0003-4719-1951

Anchalee Aowphol https://orcid.org/0000-0001-9504-4601

### Data availability

All of the data that support the findings of this study are available in the main text or Supplementary Information.

## Supplementary materials

Supplementary material 1 Material examined in the molecules, locality, collection numbers, GenBank accession numbers and references Data type xlsx Explanation note Material examined, including locality, collection numbers, GenBank accession numbers, and references for the specimens used in the phylogenetic analyses. https://binary.pensoft.net/file/1127579This dataset is made available under the Open Database License (http://opendatacommons.org/licenses/odbl/1.0/). The Open Database License (ODbL) is a license agreement intended to allow users to freely share, modify, and use this Dataset while maintaining this same freedom for others, provided that the original source and author(s) are credited.Natee Ampai, Attapol Rujirawan, Siriporn Yodthong, Korkhwan Termprayoon, Bryan L. Stuart, Anchalee Aowphol

Supplementary material 2 Data for multivariate analyses of the *Cyrtodactylusintermedius* group Data type xlsx Explanation note Dataset of morphological and meristic characters for multivariate analyses of the *Cyrtodactylusintermedius* group. https://binary.pensoft.net/file/1127580This dataset is made available under the Open Database License (http://opendatacommons.org/licenses/odbl/1.0/). The Open Database License (ODbL) is a license agreement intended to allow users to freely share, modify, and use this Dataset while maintaining this same freedom for others, provided that the original source and author(s) are credited.Natee Ampai, Attapol Rujirawan, Siriporn Yodthong, Korkhwan Termprayoon, Bryan L. Stuart, Anchalee Aowphol

Supplementary material 3 Mean uncorrected p-distances (%) within the *Cyrtodactylusintermedius* group based on the mitochondrial ND2 gene and flanking tRNAs Data type xlsx Explanation note Mean (minimum–maximum) uncorrected p-distances (%) within the *Cyrtodactylusintermedius* group based on 1,227 bp of the mitochondrial ND2 gene and flanking tRNAs. Numbers in bold are within species divergence. n = number of individuals. https://binary.pensoft.net/file/1127581This dataset is made available under the Open Database License (http://opendatacommons.org/licenses/odbl/1.0/). The Open Database License (ODbL) is a license agreement intended to allow users to freely share, modify, and use this Dataset while maintaining this same freedom for others, provided that the original source and author(s) are credited.Natee Ampai, Attapol Rujirawan, Siriporn Yodthong, Korkhwan Termprayoon, Bryan L. Stuart, Anchalee Aowphol

Supplementary material 4 Summary of morphological characters, meristic characters, and color pattern data of the *Cyrtodactylusintermedius* group Data type xlsx Explanation note Summary of morphological characters, meristic characters, and color pattern data of the *Cyrtodactylusintermedius* group. Abbreviations are defined in Materials and methods. Key: NA = no data or not applicable. https://binary.pensoft.net/file/1127582This dataset is made available under the Open Database License (http://opendatacommons.org/licenses/odbl/1.0/). The Open Database License (ODbL) is a license agreement intended to allow users to freely share, modify, and use this Dataset while maintaining this same freedom for others, provided that the original source and author(s) are credited.Natee Ampai, Attapol Rujirawan, Siriporn Yodthong, Korkhwan Termprayoon, Bryan L. Stuart, Anchalee Aowphol
